# Persistence of Livestock Associated MRSA CC398 in Humans Is Dependent on Intensity of Animal Contact

**DOI:** 10.1371/journal.pone.0016830

**Published:** 2011-02-09

**Authors:** Haitske Graveland, Jaap A. Wagenaar, Kelly Bergs, Hans Heesterbeek, Dick Heederik

**Affiliations:** 1 Division Environmental Epidemiology, Institute for Risk Assessment Sciences, Utrecht University, Utrecht, The Netherlands; 2 Department of Infectious Diseases and Immunology, Faculty of Veterinary Medicine, Utrecht University, Utrecht, The Netherlands; 3 Department of Farm Animal Health, Faculty of Veterinary Medicine, Utrecht University, Utrecht, The Netherlands; 4 Central Veterinary Institute of Wageningen UR, Lelystad, The Netherlands; 5 Julius Center for Health Sciences and Primary Care, University Medical Center, Utrecht, The Netherlands; University of Oxford, Viet Nam

## Abstract

**Introduction:**

The presence of Livestock Associated MRSA (LA-MRSA) in humans is associated with intensity of animal contact. It is unknown whether the presence of LA-MRSA is a result of carriage or retention of MRSA-contaminated dust. We conducted a longitudinal study among 155 veal farmers in which repeated nasal and throat swabs were taken for MRSA detection. Periods with and without animal exposure were covered.

**Methods:**

Randomly, 51 veal calf farms were visited from June - December 2008. Participants were asked to fill in questionnaires (n = 155) to identify potential risk factors for MRSA colonisation. Nasal and throat swabs were repeatedly taken from each participant for approximately 2 months. Swabs were analysed for MRSA and MSSA by selective bacteriological culturing. *Spa*-types of the isolates were identified and a ST398 specific PCR was performed. Data were analyzed using generalized estimation equations (GEE) to allow for correlated observations within individuals.

**Results:**

Mean MRSA prevalence was 38% in farmers and 16% in family members. Presence of MRSA in farmers was strongly related to duration of animal contact and was strongly reduced in periods with absence of animal contact (−58%). Family members, especially children, were more often carriers when the farmer was a carrier (OR = 2, P<0.05).

Only 7% (n = 11) of the participants appeared to be persistent carriers. A large heterogeneity in *spa*-types was detected, however 92.7% belonged to LA-MRSA CC398. A surprisingly high fraction of the spa-types (7.3%) did not belong to CC398.

**Conclusion:**

The presence of LA-MRSA in farmers is strongly animal-exposure related. The rapidly decreasing MRSA prevalence during absence of animal contact suggests that LA-MRSA is a poor persistent colonizer in most humans. These results are of relevance for MRSA control strategies.

## Introduction

Infections with Methicillin-Resistant *Staphylococcus aureus* (MRSA) are associated with increased morbidity and mortality, length of hospitalization and health care costs [1], [2]. Surveillance data of MRSA in The Netherlands and Scandinavian countries showed that MRSA prevalence is low (<1%), whereas the prevalence in some other European countries has reached values up to 50% [3]. The low prevalence in The Netherlands and Scandinavian countries in hospitals is maintained by an active “Search and Destroy” policy and restrictive antibiotic use in human healthcare. Patients with increased risk for MRSA colonization are screened at hospital admission, and cared for in isolation. Furthermore, specific hospital hygiene measures have been implemented [2], [4]. This approach is costly for the health care system, but considered cost-effective.

Since 2003, MRSA belonging to Clonal Complex (CC) 398 (CC398) has emerged in livestock and this CC is by far the most prevalent livestock-associated MRSA (LA-MRSA). CC398 is now being reported from different countries around the world [5], [ 9]–[11]. The emergence in livestock caused a strong increase in MRSA occurrence in humans between 2001 and 2006 in The Netherlands [8] [12]. Identification of risk factors and knowledge about persistence of LA-MRSA in humans is essential for successful continuation of the Search and Destroy strategy. We recently observed a high prevalence of MRSA in veal farmers (∼30%) and their family members (<10%). In particular, intensity of animal contact and MRSA occurrence among calves were risk factors for MRSA colonization in humans [13].

Studies indicate that carriage of MRSA of hospital origin may persist for several months up to years [16], although available studies had limited power and cannot be generalized easily because they involve specific patient populations [14], [ 15]. The proportion of long-term carriers (> than 1 year) ranges between 10–20% [1], [ 14], [ 17], [ 18]. Currently, no data are available about persistence and dynamics of MRSA CC398 carriage and the possible role of intensity of contact with livestock. Understanding the dynamics of MRSA carriage in farmers occupationally exposed to MRSA is essential in designing specific control strategies. The aim of this longitudinal study was to determine the persistence and dynamics of MRSA carriage in individuals in close contact with veal calves in periods with and without animal exposure.

## Materials and Methods

### Study design an study population

**Figure 1:**
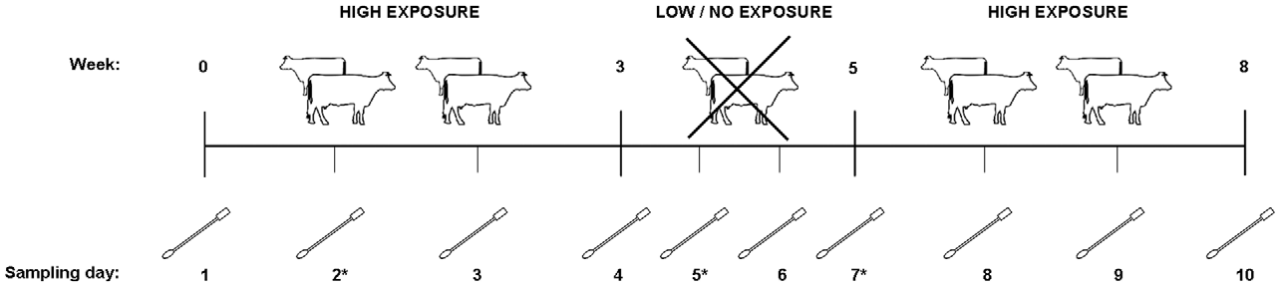
Schematic overview of the study design. Participants were followed for approximately two months during periods of both high and low/no exposure. The study starts 3 weeks prior to, and ends 3 weeks after, the low/no exposure period. Nasal and throat swabs were taken in the morning and evening once a week in high exposure periods, and twice a week in low exposure period. * Nasal samples taken in the evening of sampling day 2 (high exposed), 5 and 7 (low exposed) were additionally screened for MSSA.

The study population consisted of 155 individuals living or working on randomly selected veal farms (n = 51) in The Netherlands. Participants included had no occupational contact with other animals than calves. Participants were followed for approximately two months between June and December 2008 during periods of both high and low or no exposure. During high exposure, veal calves were present on the farm. During low exposure, participants were on a holiday (no exposure), or animals were absent in-between production cycles (low exposure). Hereafter, ‘low exposure’ is used when a participant had a holiday or an empty barn period. The study period started three weeks prior to low exposure (when calves were still at the farm) and ended three weeks after this period (when the farm was populated with new calves) (Figure 1).

Participants were trained in taking swabs and asked to take nasal and throat swabs in the morning (before animal contact when present) and evening (after animal contact when present). Participants were alerted by phone text messages or e-mail for prompt timing. Dry swabs were taken weekly during high exposed periods, and twice a week during low exposed periods. On average, each participant was sampled on 10 days. Swabs were sent to the laboratory by mail. Questionnaires were used to register risk factors including farm characteristics, time spent on the farm, hygiene practices, and if available, MRSA anamneses, as well as potential confounders like age, gender and smoking habits. A short questionnaire was used to collect information related to specific sampling days (activities, duration of animal contact, contact with other animals than calves) and the three days before sampling. From these questionnaires the duration of animal contact was defined as the sum of time periods for all activities involving animal contact (feeding calves, veterinary care and working in the calf stables in general) in the three days before sampling. The duration of the low exposed period is defined as the duration in days of the period that either no animals were present on the farm or participants were on holiday.

The study protocol was approved by the Medical Ethical Committee of University Medical Centre, Utrecht. All participants completed a written informed consent form.

Participants were considered MRSA positive and defined as “carrier” when MRSA was isolated in at least one out of four samples per day. We will use the word “carriage” or “carrier” for these individuals from here on. Persistent carriage was defined as having MRSA positive results (i.e. testing positive for MRSA presence) on all sampling days. Intermittent carriage was defined as having fewer than ten, but more than zero, positive days. Individuals with only MRSA negative swab samples were defined as non-carriers.

### Laboratory analyses

All samples were analysed as previously described [19]. Briefly, swabs were inoculated in a non-selective pre-enrichment containing Mueller Hinton broth with 6.5% NaCl. After overnight aerobic incubation at 37°C, 1 mL of pre-enrichment was transferred into 9 mL selective enrichment broth (BioMérieux, France). Ten µL of this selective enrichment broth was inoculated onto sheep blood agar (Biotrading, The Netherlands) and MRSA BrillianceTM agar (Oxoid, The Netherlands). All suspected colonies were identified as *S*. *aureus* using standard techniques: colony morphology and coagulase assay (slide). The presence of the *mecA*-gene was confirmed by PCR [20]. A random selection of strains (n = 478) was confirmed by *S. aureus* specific PCR [21].

Nasal samples taken in the evening of sampling day 2 (high exposed), day 5 and day 7 (low exposed) were also analysed for the presence of MSSA. Ten µL of the pre-enrichment broth were inoculated onto SA-Select agar (BioRad, The Netherlands), a *S. aureus* specific medium. Suspected colonies were identified as *S*. *aureus* using standard techniques: colony morphology, coagulase assay (slide) and by Pasteurex Staph-plus (Bio-Rad). The absence of the *mecA*-gene was confirmed by PCR [20]. A random selection of the MSSA strains (n = 104) was confirmed by PCR for the *S. aureus* specific DNA-fragment Martineau [21].

*Spa*-types were determined for one MRSA (n = 375) and one MSSA (n = 104) nasal isolate per sampling day and in addition for all throat samples (n = 103) (MRSA). The strains were *spa-*typed by sequencing of the repetitive region of the protein A gene *spa* [22]. A random selection of five strains per *spa*-type was tested with CC398 specific PCR [23].

### Data analysis

Statistical analyses were conducted using SAS software 9.1 (SAS Institute, Inc., Cary, NC). Generalized estimation equations (GEE) were used to study associations of MRSA carriage in nose and throat with determinants, adjusting for correlations between repeated measures in the same individual and clustering of individuals by farm. Analyses were performed for nose and throat samples both, separately, and combined. Age, gender and smoking were included in the model as fixed effects, exposure was time varying. A P-value of <0.05 was considered statistically significant.

## Results

### MRSA carriage

Nasal (n = 2864) and throat (n = 2865) swabs were taken from 155 individuals. The response was 91%. Reasons given for non-participation were no interest or lack of time. The low exposure period involved a holiday for 38 participants and a period between production cycles for 117 participants (Table 1). Mean MRSA prevalence over the study period was 38% in farmers and 16% in family members. Family members were more often MRSA carriers when the farmer was an MRSA carrier, even after adjustment for the number of hours working in the calf stable in the 3 days before sampling (OR = 2.0, P<0.05). Stratified analysis for both partners (spouses) and children showed comparable point estimates for the effect of MRSA carriage by the farmer, but the association was not statistically significant for partners. The effect of MRSA carriage of the farmer on carriage risk in family members was not statistically significant in subsets of the sampling data and could only be picked up when the complete dataset was used.

**Table 1:** Characteristics of study population of 155 participants.

	Number of participants (%) (n = 155)
**Gender**MaleFemale	88 (57)67 (43)
**Category**FarmerPartner of farmerChild of farmerEmployees	51 (33)40 (26)57 (36.5)7 (4.5)
**Age**Age (mean, range)age <18age 18–65age >65	155 (33, 1–79)40 (26)110 (71)5 (3)
**Smoking**	32 (21)
No Smoking	123 (79)
**Exposure**	
Number of hours working in calf stable in 3 days before sampling (mean, (range))<6 hours>6 hours	155 (6, (0–49))109 (70)46 (30)
**Duration of low exposure period in days (mean (range))**Duration of empty barn period in days (low exposure) (mean,(range))<14 days>14 daysDuration of holiday period in days (no exposure) (mean,(range))<10 days>10 days	155 (13, (3–32))117 (14, 3–32)63 (41)54 (35)38 (11, 4–21)16 (10)22 (14)

MRSA prevalence was strongly reduced during low exposure periods. The reduction was strongest for holiday periods; during holiday periods the prevalence was 11% compared to 26% in exposed periods (−58%). The average prevalence of MRSA during low exposed periods, was reduced from 25% to 19% (−24%) (Table 2).

**Table 2:** Mean daily MRSA and MSSA prevalence.

	Mean prevalence, % (95% CI∧)
**MRSA Carriage**Nasal MRSA carriage (total n = 155)*Throat MRSA carriage (total n = 155)*High exposed (total n = 155)**High exposed (holiday n = 38)**High exposed (empty barn n = 117)**Low exposed (total n = 155)**No exposed (holiday n = 38)**Low exposed (empty barn n = 117)**	16.7 (14.7–18.5)3.6 (2.7–4.6)24.7 (22.2–27.4)25.6 (18.4–30.4)24.4 (21.7–27.6)19.0 (14.9–23.1)11.1 (4.1–18.1)21.7 (16.3–25.9)
**MSSA carriage** (total n = 155)***	23.0 (19.1–26.9)

∧ CI, confidence interval.

* mean prevalence over 10 sampling days based on nasal/throat swabs taken in morning.

** mean prevalence over sampling days in either low (on average 3.4 sampling days) or high exposed period (on average 5.8 sampling days).

*** mean prevalence over 3 sampling days based on nasal swabs taken in evening.

MRSA carriage was less frequently seen in throat samples compared to nasal samples (Table 2, p<0.05). Farmers who worked more hours in stables on days before sampling were more often carriers compared to individuals who worked fewer hours (Odds Ratio (OR)  = 1.5, (P = 0.03) expressed per 3 working days of 8 hours each). The longer farmers were non-exposed, the lower MRSA carriage prevalence was (OR = 0.5, (P = 0.01) for those exposed above and below median duration of 12 days; Table 3), both for nasal and throat carriage. Potential confounding variables like age, gender and smoking habits had associations in the expected direction but hardly changed associations between MRSA and duration of exposure; age was positively associated (OR = 1.2 (P = 0.04) per 10 years) and males were significantly more often MRSA colonized compared to females (OR = 1.9, (P = 0.04)). Smoking was negatively associated (OR = 0.6 (P = 0.22)), although not statistically significantly so (Table 3).

**Table 3:** Associations between MRSA nasal and throat carriage and determinants in a multiple regression analysis.

	Nasal swabs (n = 2804)OR (95% CI)	Throat swabs (n = 2805)OR (95% CI)	Nasal and Throat swabs (n = 5609)OR (95% CI)
Age**	1.4 (1.1–1.7)*	1.1 (1.1–1.7)	1.2 (1.1–1.4)*
Gender (ref ♀)	3.4 (1.7–6.6)*	1.3 (1.7–6.6)	1.9 (1.1–3.4)*
Smoking(ref no smoking)	0.4 (0.1–1.0)*	0.6 (0.1–1.0)	0.6 (0.3–1.3)
Duration of Low exposed period***(ref: above median duration of 12 days)	0.5 (0.12–1.3)	0.4 (0.2–1.3)*	0.5 (0.3–0.9)*
Duration of animal contact in 3 days before sampling****	1.7 (1.1–2.8)*	2.2 (1.1–4.5)*	1.5 (1.1–2.2)*

* p<0.05,

** Expressed per 10 years,

*** Refers to duration in days of either empty barn (low exposure) or holiday period (no exposure),

**** Expressed per 24 hours. Refers to duration of exposure to animals in 3 days before sampling.

### Persistence or MRSA carriage

Because MRSA carriage was strongly associated with duration of animal contact, large fluctuations in carrier status over time were seen (Tables 3, 4, 5). Only 11 participants (7%), who were mainly farmers (n = 9), were MRSA positive throughout the study period at each sampling point (Tables 4 and 5) and were therefore defined as persistent carriers. Only one persistent carrier had a holiday, while the others still lived on the farm during a low exposure period when animals were absent. The majority of the study population (n = 93) were intermittent carriers (58%). Fifty-four participants tested always MRSA negative (35%) (Table 5). The prevalence of MRSA carriage in the throat was clearly higher in persistent carriers (35.3%) as compared to intermittent carriers (3.6%) (Table 5). The majority of persistent carriers were MRSA positive in their nose. One participant was MRSA negative in the nose during one out of ten sampling days. However, the throat sample of this person turned out to be MRSA positive on this day. Only one individual was a persistent MRSA carrier on the basis of the throat swabs only. All other persistent carriers were nasal carriers only, or were persistent MRSA carriers in both nose and throat.

**Table 4:** Persistence of MRSA carriage in farmers, family members and others.

Number of MRSA positive sampling days*	0	1	2	3	4	5	6	7	8**	9**	10**	Total
Farmer	13	13	1	2	4	1	3	2	3+**2**	**2**	**5**	51
Partner of farmer	11	14	6	3	3	1	0	0	0	0	**2**	40
Child of farmer	25	17	7	3	0	1	3	0	1	0	0	57
Others	5	0	1	0	0	0	1	0	0	0	0	7
Total	54	44	15	8	7	3	6	2	6	2	7	155

* MRSA positive means MRSA detected in at least one out of 4 samples (nasal and/or throat) taken per sampling day.

** Persistent carriers in bold (Persistent carrier: MRSA positive results on all sampling days; number of sampling days dependent on duration of low exposed period).

**Table 5:** Characteristics of persistent-, intermittent- and non-carriers of MRSA.

	MRSA* nasal carriage(Days positive/total days sampled)	MRSA* throat carriage(Days positive/total days sampled)	MSSA** carriage (Days positive/total days sampled)	Mean number of working hours in calf stable in 3 days before sampling (range)	Mean number of working hours in calf stable/week (range)
Persistent carriers (n = 11/155 (7%))	102/103 (99%)	36/102 (35.3%)	0/29 (0%)	12.6 (0–40)	35.2 (0–65)
Intermittent carriers (n = 90/155 (58%))	209/835 (25.0%)	47/835 (5.6%)	76/261 (29.1%)	5.2 (0–49)	15.8 (0–65)
Non-carriers (n = 54/155 (35%))	0/490 (0%)	0/491 (0%)	27/157 (17.2%)	4.7 (0–44)	12.7 (0–70)

* mean prevalence over 10 sampling days.

** mean prevalence over 3 sampling days.

### MSSA

In total, 447 swabs of 155 participants were analyzed for presence of MSSA. A mean prevalence for MSSA of 23% was found. MSSA was found on all three sampling days in the swabs of 16 individuals (10%). MSSA was absent in the swabs of the majority of the population (64%). Forty individuals (25%) were once or twice MSSA positive out of 3 sampling days. Although not statistically significant, an inverse association was observed between MRSA carriage and MSSA carriage, in both nasal and throat swabs, adjusted for age, gender and smoking habits (Table 6). We found no MSSA carriers among persistent MRSA carriers (Table 5).

**Table 6:** Associations between MRSA carriage and determinants, including MSSA nasal carriage in a multivariate analysis.

	Nasal swabs (n = 431)OR (95% CI)	Throat swabs (n = 431)OR (95% CI)	Nasal and Throat swabs (n = 862)OR (95% CI)
Age***	1.3 (1.0–1.6)	1.1 (0.7–1.7)	1.2 (0.9–1.5)
Gender (ref ♀)	6.5 (2.5–17.2)	1.8 (0.4–7.9)	4.4 (1.9–10.0)*
Smoking(ref no smoking)	0.7 (0.2–2.0)	0.2 (0.0–2.1)	0.6 (0.2–1.5)
Duration of Low exposed period****(ref: above median duration of 12 days)	0.7 (0.2–2.0)	0.8 (0.2–3.5)	0.8 (0.3–1.8)
Duration of animal contact in 3 days before sampling*****	1.8 (0.6–5.5)	3.9 (0.7–21.0)	1.9 (0.4–4.6)
MSSA carriage	0.6 (0.2–1.6)	0.3 (0.0–2.3)	0.7 (0.3–1.6)

Analysis based on samples taken in the evening of sampling day 2, 5 and 7. Associations shown for different dependent variables separately (nasal and/or throat carriage).

* p<0.05,

** p<0.10,

*** Expressed per 10 years,

**** Refers to duration in days of either empty barn (low exposure) or holiday period (no exposure),

***** Expressed per 24 hours. Refers to duration of exposure to animals in 3 days before sampling.

### Genotyping MRSA and MSSA

*Spa-*types were determined of 375 nasal MRSA isolates and 103 MRSA throat isolates. In total 26 different *spa*-types were identified. The majority of the *spa*-types identified belonged to CC398 (92.7%). All persistent carriers were solely CC398 carriers, however we observed that in these carriers different spa-types within CC398 could be present on different sampling days. Thirty five strains (7.3%) were identified as non-CC398 types. We found no association between carriage of non-CC398 carriage and more general risk factors for MRSA such as hospital admission or travelling in a high prevalent MRSA country. No persistent non-CC398 carriers were identified. Overall, the prevalence of non-CC398 carriage was 2% per sampling day.

In addition *spa*-types of 104 MSSA isolates were determined. Out of 24 different MSSA *spa*-types the majority of the strains (56.2%) did not belong to the CC398 lineage. In 13 individuals, MRSA and MSSA were found in the same nasal swab. In 10 of these individuals, the MRSA and MSSA strains found in those nasal samples belonged to similar *spa*-types.

## Discussion

This study shows that persistence of MRSA carriage in farmers is associated with duration of animal contact. LA-MRSA prevalence drops during a low exposure period and this is strong evidence for a relation with animal exposure. The large difference in MRSA prevalence between farmers and family members and the observation that MRSA carriage is lower after a longer period of low exposure are both in line with the hypothesis that exposure to MRSA-positive animals plays a major role in MRSA carriage in farmers. The positive association between carriage status of children and MRSA carriage by the farmer, however, suggests that MRSA carriage in children might be determined more strongly by contact with highly exposed people (farmer) than by animal contact. This might be a result of the fact that on average children are less exposed to animals and therefore the effect of exposure is smaller compared to the farmers or their partners.

Furthermore, we found that the majority of the study population were intermittent carriers (58%) or non-carriers (35%), and only 11 individuals (7%) were persistent carriers. These results indicate that carriage of MRSA in a highly-exposed population is mainly transient, or that there is retention of MRSA-containing dust in the nasal cavities in absence of colonization [13]. Retention of dust leading to presence of MRSA in nasal cavities seems likely, given the exposure to high levels of dust associated with animal husbandry [24]. In addition, strong correlations have been found earlier in studies exploring associations between MRSA positive air samples and presence of MRSA colonized patients [25]. The role and function of the anterior nares in humans [26], the region of the respiratory tract where MRSA is predominant and where dust particles are deposited, supports this hypothesis.

The few available studies for Hospital Acquired MRSA (HA-MRSA) indicate long-term colonization rates over 20% [1], [ 14], [ 15], [ 17], [ 18]. Thus HA-MRSA seems a more persistent colonizer than LA-MRSA, which depends on several host characteristics and also on specific staphylococcal factors [27], [ 28], suggesting that LA-MRSA is a poor persistent colonizer in humans.

Apart from one individual, all persistent carriers had an empty barn period (low exposure) instead of a holiday period (no exposure). Some exposure may have occurred when the barn was empty. As a result, this might have affected the number of persistent carriers observed. At the start of the study we assumed that all participants were potentially exposed to MRSA because MRSA is present on the majority of the Dutch veal farms (90%) [13]. Furthermore from our previous cross-sectional study it is shown that there is significant (but not perfect) concordance between MRSA carriage in veal calves and environmental sampling using wipes (data not shown). However, testing MRSA status of a farm by taking five wipe samples from stables at the beginning and end of the study, analysed for presence of MRSA, showed that on three farms MRSA was not detected at the beginning and end of the study period. Removal of the participants of these farms from the data analysis changed the results only marginally.

The anterior nares are considered to be the primary colonization site of *S. aureus* [17]. Additional screening of the throat for presence of MRSA may lead to a significantly increased carrier prevalence [29], [ 30]. Such an effect was not observed in this study. The throat may yield fewer organisms than an anterior nare sample, either because the sampling technique is sub-optimal for the throat, or because there are fewer organisms present at the sampling site [30]. If exposure plays a major role in screening MRSA in a population of farmers, for example because of deposition of MRSA-containing dust in the nasal cavities, it may be the case that that throat samples provide a more reliable estimate of the MRSA-carriage status compared to nasal samples. However, inhaled and deposited dust is removed from nasal cavities by cilliary transport and subsequent swallowing. Consequently, a gold standard for MRSA screening and detection is not available.

None of the persistent carriers received antibiotics over the year prior to the study. Thus an increase in throat-carriage due to antibiotic treatment associated shift in carrier site could not have occurred [31]. Environmental contamination with penicillin or tetracycline might be an important risk factor for colonization of the nares, and contributes to further spread in hospital patients [32], [ 33]. Environmental contamination with antibiotics might have occurred in stables where animals receive antibiotics. Residues of this antibiotic substance occur in manure, in air or on surfaces of animal housing [34]. This may have contributed to the high MRSA nasal prevalence in farmers, but this is in need of further exploration.

Observational studies which investigate risk factors of LA-MRSA in humans are mainly based on a single measurement of one nasal swab [5], [ 6]. The present study shows considerable variability in LA-MRSA carriage over time. Guidelines underlying the Search and Destroy policy have been adapted due to conclusions from observational studies based on single nasal swabs. Pig and veal calf farmers are defined as new risk populations for MRSA carriage and are actively screened when admitted to a hospital [2]. The substantial increase in health-care costs due to the presence of LA-MRSA, could be reduced by changing control measures to include an exposure-free period for farmers before screening. In addition, treatment of positive farmers is not meaningful when accompanied by ongoing exposure. Treating people only in the absence of exposure will limit the number of antibiotic treatments necessary for a successful MRSA decolonization compared to continuously exposed people and therefore contributes to a restrictive antibiotic-use policy.

Other studies suggested that carriage with *S. aureus* might have a protective effect on the acquisition of other strains [17], [ 35], possibly through colonization competition [35]. The negative association between MSSA and MRSA carriage found in this study also suggests that competition might play a role. So far, studies where risk factors for LA-MRSA colonization were investigated did not take the possible interference between *S. aureus* strains into account. In this population 64% of the participants were non-carriers, 25% were intermitted carriers and 10% were persistent MSSA carriers. These figures differ from what is generally found in hospital settings. The number of persistent and intermittent carriers there is generally higher (20% persistent – 60% intermittent carrier) and the number of non-carriers is considerably lower (20% almost never carry *S. aureus*) [17]). Because exclusive throat carriage of MSSA is also reported [29], [ 30], it is possible that we underestimate the MSSA prevalence and number of persistent and intermittent carriers. Screening for MSSA using *S. aureus-*selective plates might also have contributed to differences, because both MRSA and MSSA may grow on these plates. It can therefore not be excluded that MSSA could have been present but was missed in samples with predominant MRSA growth.

This study showed a variety in CC398 spa-types and an unexpected relatively large number of non-CC398 strains were isolated. These strains could not be related to known MRSA risk factors, such as visits to health care facilities. This is remarkable, because, so far, MRSA *spa*-types other than CC398 are seldom seen in veal farmers [13]. Co-colonization of CC398 and non-CC398 may potentially lead to exchange of properties, e.g. improved transmission of CC398. Although results of this study indicate limited public health risk of LA-MRSA carriage, it still remains important from a public health point of view to control spread of LA-MRSA because of this potential for adaptation.

### Conclusions

The present study indicates that carriage of LA-MRSA in farmers is strongly exposure related and mainly transient. It suggests that LA-MRSA is a poor persistent colonizer in humans. Improved understanding of the role of exposure and host specificity of LA-MRSA could have a significant impact on antibiotic and infection control policies in the hospitals, and more importantly, on the development of new strategies for the control of MRSA.
